# Effects of Dark Septate Endophytes Strain A024 on Damping-off Biocontrol, Plant Growth and the Rhizosphere Soil Enviroment of *Pinus sylvestris* var. *mongolica* Annual Seedlings

**DOI:** 10.3390/plants9070913

**Published:** 2020-07-20

**Authors:** Xun Deng, Xiaoshuang Song, Saiyaremu Halifu, Wenjing Yu, Ruiqing Song

**Affiliations:** 1 Institute of Forestry Protection, Heilongjiang Academy of Forestry, Harbin 150040, China; dxhappy@126.com (X.D.); sxshappy@126.com (X.S.); ywjlinda2008@163.com (W.Y.); 2 College of Forestry, Northeast Forestry University, Harbin 150040, China; saiyaremu@nefu.edu.cn

**Keywords:** dark septate endophytes (DSEs), *Pinus sylvestris* var. *mongolica*, damping-off biocontrol, growth stimulation, microecological environment in soil

## Abstract

Dark septate endophytes (DSEs) exert a vital role in promoting plant growth, improving mineral absorption, biological disease control, and enhancing plant stress resistance. The effects of dark septate endophyte strain, *Phialocephala bamuru* A024 on damping-off biocontrol, plant development, nutrients within the rhizosphere soil, as well as bacterial communities in the annual seedlings of *P. sylvestris* var. *Mongolica* were studied. According to our findings, following *P. bamuru* A024 inoculation, the damping-off disease morbidity decreased significantly compared with control, some physiological indices such as β-1,3-glucanase, chitinase enzyme activity as well as a soluble protein and proline content in *P. sylvestris* var. *mongolica* were elevated under *R. solani* stress. After inoculation with *P. bamuru* A024, the biomass in seedlings, nutrients in soil, root structure index, together with activities of soil enzymes were remarkably up-regulated relative to control (*p* < 0.05). As suggested by the results of high-throughput sequencing, the microbial structure in the rhizosphere soil of the *P. sylvestris* var. *mongolica* showed significant differences (*p* < 0.05) after *P. bamuru* A024 inoculation compared to control treatment and the rhizosphere soil bacterial community structure after DSE A024 inoculation was positively correlated to the main soil nutrition indices.

## 1. Introduction

Plant endophytes widely exist in plants [1]. Dark septate endophytes (DSE) represent a major group on endophytes within plants characterized by a dark mycelium color and distinct septum. They colonize the epidermis, cortex, and even the intercellular space of vascular tissue of healthy plant roots to form symbionts without causing plant diseases [2,3]. The host range of dark septate endophytic fungi covers nearly 600 species of plants from 114 families and 320 genera. DSE colonization has been found in mycorrhizal plants as well as roots of traditional non-mycorrhizal plants such as Cyperaceae, Cruciferae, and Chenopodiaceae [4]. In conifers, the main DSE fungi are the group of the *Phialocephala fortinii* s.l.—*acephala applanata* species complex (PAC) belonging to the ascomycetes. PACs can be found in the Northern Hemisphere from polar to tropical areas and play a leading role in the root system of conifers [5,6]. Several studies have reported that DSE fungi exhibit a positive effect on plant growth [7,8,9,10]. They enhance the mineralization process of insoluble phosphorus in soil [11,12], promote the uptake and utilization of nutrients like nitrogen (N) or phosphorus (P) by the plants [13] and increase the stress tolerance of the host plant [14,15,16,17]. In addition, DSE fungi can colonize the host roots and effectively inhibit the occurrence of soil-borne diseases [18,19,20,21].

Mongolian pine (*P. sylvestris* var. *mongolica*) is a geographical variety of the Scots pine (*P. sylvestris*). Its natural habitats are the Daxinganling mountains in China and parts of Russia and Mongolia [22]. Due to its advantages of rapid growth, cold tolerance, drought resistance, and potent adaptability [23], it is now the major coniferous tree species used for the “Sand-Control Project” in China, which has a vital role in environmental restoration and ecological conservation. Damping-off is the most widespread and destructive disease that affects young conifer seedlings in forest nurseries around the world [24] Several soil-borne pathogens such as different species of *Pythium*, *Fusarium*, and *Rhizoctonia* coexist in most nursery soils and are responsible for severe damage in nurseries [25]. Damping-off damage caused by *Rhizoctonia*, particularly *R. solani*, is widely reported in forest nurseries around the world [26,27,28]. Like other conifer seedlings, the soil-borne diseases of *P. sylvestris* var. *Mongolica* are managed in the nursery by chemical control. However, pesticides and chemical fertilizers have often been excessively applied, which leads to a variety of negative impacts, like critical illness, damage to the soil environment, or retarded seedling growth [29]. At present, the use of microbial metabolites produced by beneficial microbiota is a novel, environmentally friendly approach to manage plant health in comparison with the use of chemical pesticides. It is advantageous in that it is free of pollution or residues, it shows pathogen specificity, difficulty in resistance generation and has human or animal health benefits, while providing environmental protection [30].

*P. fortinii* s.l.—*acephala applanata* species complex (PAC) members have been noted to suppress pathogens [31]. *P. sphareoides* could effectively inhibit *Heterobasidion parviporum* infection of Norway spruce by producing a variety of bioactive substances, as well as reduce mortality and the disease intensity [32]. PAC strains with low toxicity were also verified by inoculation experiments to control high toxicity PAC pathogens using niche competition [33]. Christoph [34] isolated 85 PAC strains from Norway spruce (*Picea abies*) roots, among which *P. europaea* dramatically decreased *Phytophthora citricola* growth in vitro. *P. bamuru* A024 (isolated from the root of *P. sylvestris* var. *Mongolica* collected on the Jiagedaqi Experimental Forest Farm in Hei Longjiang Province, China) is one of the PAC members that has a strong ability to inhibit the growth of *R. solani* in vitro (unpublished data). In this study, *P. bamuru* A024 was utilized for investigating the impacts of DSEs on damping-off biocontrol in vivo, root structure and growth promotion for the *P. sylvestris* var. *Mongolica* annual seedlings, rhizosphere soil physicochemical characters, as well as the microbial community structure. Thus, our study aimed to assess the impacts of *P. bamuru* A024 on: (1) Seedling damping-off disease of *P. sylvestris* var. *mongolica*; (2) growth promotion as well as root structure in the seedlings in *P. sylvestris* var. *mongolica*; (3) rhizosphere soil physical characteristics together with enzymatic activities of the seedlings in *P. sylvestris* var. *mongolica*; (4) rhizosphere microbial structure in the seedlings of *P. sylvestris* var. *mongolica*.

## 2. Materials and Methods

### 2.1. Organisms and Growing Environment

Dark septate endophytes strain, *P. bamuru* A024 (NCBI accession number: MN006137), was isolated from the root of *P. sylvestris* var. *Mongolica*, collected at the Jiagedaqi Experimental Forest Farm in Hei Longjiang Province of China. The pathogenic fungus, *R. solani* SH01, was isolated from roots of diseased conifer seedlings, collected at the Weihe seedling nursery of Hei Longjiang Province, China. The above two strains were then inoculated on potato dextrose agar (PDA) medium supplemented with 12 g/L potato extract, 20 g/L dextrose and 14 g/L agar (pH 6.0, Haibo Biotechnology, Qingdao, China).

After 20 days of growth on PDA medium, *P. bamuru* A024 mycelium was collected using a 5 mm sterile puncher. The mycelia disks were transferred to solid cottonseed shell medium [35] (cottonseed shell 200 g, glucose 2 g, MgSO_4_·7H_2_O 3 g, KH_2_PO_4_ 3 g, (NH_4_)_2_HPO_4_ 3 g, Ca_3_(PO_4_)_2_ 2 g, vitamin B_1_ 1 mg, 60% water content, and steam-sterilized at 0.1 MPa at 121 °C for 1 h), followed by 30 days of incubation at 25 °C in the dark.

The pathogenic fungi, *R. solani* SH01, after five days of growth on the PDA medium, was sampled using the 5 mm sterile puncher. A suspension of *R. solani* SH01 was prepared by placing the mycelium inoculant in liquid PD medium (agar-free PDA medium) and incubating for 7 days under 25 °C in dark with 150 rpm of agitation. The culture suspension was mixed with sterile culture substrate (vermiculite:river sand:peat soil = 1:1:3, v:v:v) in a ratio of 1:10 (v:v), and subsequently used for seedling inoculation [35].

Potassium permanganate (0.5% v/v) was used to sterilize the surface of *P. sylvestris* var. *mongolica* experimental seeds (Zhanggutai Experimental Forest Farm, Zhangwu County, Liaoning Province, China) for 30 min, later, distilled water was used to rinse the seeds five times. Thereafter, seeds were further subjected to 5 days of germination on sterilized wet gauze at 25 °C.

A mixture consisting of peat soil, sand and vermiculite in a volume ratio of 2:1:1 was used as the substrate for culturing. First it was disinfected for 2 h in a high-temperature autoclave at 121 °C, and after cooling, it was transferred into plastic pots (15 cm × 13 cm). Each pot was first filled with 600 g culture substrate followed by 100 g of solid inoculum (for the control treatment, the same amount of sterile cottonseed shell culture medium was used), ensuring the coverage using the culture substrate was 1 cm in thickness. Germinated seeds (30 seeds per pot) were covered with 2 cm thick culture substrate, and then the substrate was watered and grown in the dark. After the seedlings emerged from the soil they were placed in a greenhouse at 10 plants per pot and 20 pots per treatment. Each treatment was divided into two groups and randomly subjected to the different treatments. The plants were watered once every two days, and Hoagland nutrient solution was poured on them once a week [23].

### 2.2. Design of Experiments and Inoculation of Seedlings

In each treatment (also for control), 20 pots (about 10 seedlings/pot) were selected so altogether 200 seedlings were prepared in each treatment. The four treatments included: (1) inoculation with sterile cottonseed shell medium (CK); (2) *P. bamuru* A024 (DSE) inoculation alone; (3) *R. solani* SH01 (CK + SH) inoculation alone; and (4) inoculation with *P. bamuru* A024 and *R. solani* SH01 (DSE + SH). One month after sowing, the seedlings of *P. sylvestris* var. *mongolica* were incubated using the pathogen (50 mL per pot), spread flat around to the seedlings. Each treatment was randomly assigned in the greenhouse environment, as mentioned previously.

### 2.3. Damping-Off Control Together with Physiological Index Determination

Damping-off rate in seedlings was investigated after 45 days of inoculation with *R. solani* SH01, and the relative control effect was also calculated. The survival rate of seedlings was counted three months after sowing. About 100 seedlings were investigated per treatment.

Forty five days after inoculation with *R. solani* SH01, 40 *P. sylvestris* var. *mongolica* seedlings were collected for the physiological index measurements. The roots of seedlings were rinsed using sterile water, then sterile filter paper was used to absorb excess water. The seedlings were further cut into 1 cm pieces, ground in liquid nitrogen and transferred to a 5 mL centrifuge tube. An acid ninhydrin colorimetry approach was applied to measure proline. The thiobarbituric acid chromogenic method was applied to measure malonaldehyde (MDA) content. Anthrone colorimetry was applied in measuring soluble sugar content. The Coomassie brilliant blue G-250 staining method was applied to measure soluble protein content. The nitroblue tetrazolium colorimetry approach was applied to measure superoxide dismutase (SOD) activity. The guaiacol method was applied to measure peroxidase (POD) activity. A hydrogen peroxide ultraviolet absorption method was applied to measure catalase (CAT) activity. The 3,5-dinitrosalicylic acid (DNS) method was applied to measure chitinase activity. The reducing sugar colorimetry method was applied to measure β-1,3-glucanase activity. The deamination of phenylalanine method was applied to measure phenylalanine ammonia lyase (PAL) activity. All the above indices were determined with detection kits (Nanjing Jiancheng Bioengineering Institute, Nanjing, China).

### 2.4. Sample Collection and Seedling Analysis

At 3 months after sowing, all seedlings were collected, and soil was carefully removed by washing so as to not damage the root system. Altogether 100 seedlings were randomized for every treatment, among which the initial 30 seedlings were adopted for measuring biomass indexes. In every seedling, the biomass indexes included plant height, dry weight, fresh weight upon collection, and ground level diameter. After measuring fresh weight, seedlings were dried in an oven for 5 h under 85 °C to measure their dry weight [36].

When sampling, after washing the root system to remove the soil trying to not damage the roots system of seedlings, 10 seedlings were randomly selected and an Epson v700 root scanner (Seiko Epson Corporation, Nagano, Japan) was used to scan and grade the root system of the seedlings. The indexes of mean diameter, root tip number, bifurcation number, root volume, root length and surface area were thus analyzed.

### 2.5. Soil Character Analysis

Soil was sampled from the place where the 100 seedlings were harvested for each treatment. Rhizosphere soil samples were obtained within the root zone at a depth of 5 mm by using a brush and then filtered by a 1-mm mesh sieve. Thereafter, those soil samples adopted for measuring enzymatic activities and determining physicochemical characters were dried in the air at 25 °C, packaged in the sterile sample bags, and finally preserved at 5 °C for later analyses.

The Kjeldahl approach was applied in measuring total nitrogen (TN) and the alkaline hydrolysis diffusion assay was conducted to determine the available nitrogen (AN). After sulfuric acid digestion, total phosphorus (TP) was determined by the anti-colorimetry of MO-SB method and total potassium (TK) was determined by flame photometry. The sulfuric acid-hydrochloric acid (double acid) extraction and anti-colorimetry of MO-SB method was applied to measure available phosphorus (AP). NH_4_OAc leaching and flame photometry was used to determine available potassium (AK). In addition, the potassium dichromate oxidation-external heating approach was employed for measuring organic matter (OM). The soil pH (1:2.5) was determined by using a pH meter [37]. The sodium phenol colorimetry approach was employed to measure urease activity. The glucose colorimetry approach was employed to measure saccharase activity. The disodium phenylphosphate colorimetry approach was employed to measure acid phosphatase activity. The hydrogen peroxide ultraviolet absorption method was applied to measure catalase activity. These four soil enzyme activities were determined using detection kits (Nanjing Jiancheng Bioengineering Institute).

### 2.6. Analysis of Bacterial Diversities

Rhizosphere soils were sampled according to the abovementioned method, then a 5.0 g sample was prepared for each biological replicate, which was put into a 50 mL sterilized centrifuge tube before it was sent to our laboratory in a cooler containing ice bags. In addition, soil that was utilized in high-throughput sequencing was preserved in a centrifuge tube at –80°C for further assessment.

Then, total genome DNA was extracted from the 0.5 g soil sample using the EZNA Soil DNA Kit (Omega Bio-Tek, Norcross, GA, USA) in accordance with manufacturer’s protocol. The sample was then subjected to high-throughput sequencing for soil microbiota. Thereafter, 100 µL elution solution was obtained from the literature to elute the obtained DNA. The DNA quality (A_260_/A_280_) and content were determined using a NanoDrop2000 spectrophotometer (Thermo Scientific, Waltham, MA, USA). All treatments were repeated three times. Meanwhile, the 16S rRNA region was subjected to high-throughput sequencing for determining the bacterial community in the soil. The V3 + V4 region in the 16S rRNA gene of bacteria was amplified using the universal 338F (5′-ACTCCTACGGAGGCAGCAG-3′) and 806R (5′-GGACTA CHVGGGTWTCTAAT-3′) primers.

The reaction system prepared for PCr consisted of 5×FastPfu buffer (4 µL), each primer (0.8 µL, 5 µM), 2.5 mM dNTPs (2 µL), bovine serum albumin (BSA, 0.2 µL), template DNA (10 ng), FastPfu polymerase (0.4 µL) together with double-distilled water (11.6 µL) in a total volume of 20 µL. Moreover, PCR was conducted by the following conditions: 3 min at 95 °C; 30 s at 95 °C, 30 s at 50 °C, 30 s at 72 °C for 27 cycles, followed by 10 min of extension at 72 °C. Later, the AxyPrep DNA Gel Extraction Kit (Axygen Biosciences, Union City, CA, USA) was used to purify the resultant products, whereas QuantiFluor-ST (Promega, Madison, WI, USA) was used for quantification. Later, those amplicons that were purified were mixed at the equimolar dose to form a single aliquot, which was used later to construct the library. An Illumina MiSeq sequencer (Majorbio Biotechnology Co., Ltd., Shanghai, China) was applied in performing sequencing. The raw fastqfu files were filtered and merged using Trimmomatic and FLASH (version1.2.11) [38,39], whereas pyrosequencing data were analyzed using UPARSE (version 7.1, http://drive5.com/uparse/). Subsequently, all sequences were classified as operational taxonomic units (OTUs) at the similarity threshold of 97%, while UCHIME (version 4.1) was used to remove chimeras [40]. For all sequences, their taxonomic annotations were analyzed using RDP Classifier (http://rdp.cme.msu.edu/) at the 0.7 confidence threshold.

### 2.7. Data Analyses

WPS 2016 (Kingsoft Corporation, Beijing, China) was adopted to process data. Differences in index of root structure, biomass of plant, soil pH, soil enzymes and chemical characteristics were analyzed through one-way ANOVA (Tukey test) by adopting IBM SPSS 22.0 (IBM Corporation, New York, NY, USA). Moreover, correlation analysis was conducted by Pearson’s approach. A difference of *p* < 0.05 indicated statistical significance.

The Mothur software (The University of Michigan, Michigan, MI, USA) was used for analyzing α-diversity indexes as well as rarefaction. At the same time, the sequencing depth was represented by the coverage index. Moreover, both Ace and Chao1 indices were adopted for describing abundances of microbiota, whereas Shannon and Simpson indices were applied in representing species richness as well as microbial community diversity. Unifrac distance was calculated to compare the bacterial β-diversity. Differences in the phylum and genus relative abundance between four treatments were analyzed through one-way ANOVA (Tukey test) by adopting IBM SPSS 22.0. Environmental factor correlation was analyzed using RDA methods. The Origin 2019b (Origin Lab Corporation, Northampton, MA, USA) software was employed for figure generation.

## 3. Results

### 3.1. Damping-Off Control and Physiological Index Determination

The incidence rate of damping-off was 28.15%, and the survival rate was 80.15% with DSE + SH treatment. Conversely, the incidence rate of damping-off with CK + SH treatment was 72.15%, and the survival rate was 33.35%. The relative control effect of *P. bamuru* A024 treatment against the damping-off disease was 60.98%. Thus, the inoculation of strain *P. bamuru* A024 can effectively control the damping-off lesions resulting from exposure to *R. solani*.

*P. bamuru* A024 and *R. solani* inoculation affected the main physiological indices in the seedlings for *P. sylvestris* var. *mongolica* (Table 1). Differences between control (CK) and *P. bamuru* A024 treatments (DSE) were statistically significant (*p* < 0.05). The chitinase, CAT and POD activity as well as soluble protein content of the seedlings from the *P. bamuru* A024 treatment were increased by 63.21%, 59.45%, 149.63% and 49.49%, separately, relative to control treatment. Following *R. solani* inoculation, differences between control (CK + SH) and *P. bamuru* A024 treatments (DSE + SH) were statistically significant (*p* < 0.05). β–1,3-Glucanase and chitinase activity, together with the contents of proline and soluble protein in seedlings from DSE + SH treatment increased by 18.41%, 92.54%, 101.27% and 30.48%, respectively, in comparison to the CK + SH treatment. However, differences in SOD, CAT, POD and PAL activity, as well as MDA and soluble sugar contents between CK + SH and DSE + SH groups were not significant.

### 3.2. Effects of Inoculation with P. bamuru A024 on the Growth of Seedlings

#### 3.2.1. Height of Seedlings

**Figure 1:**
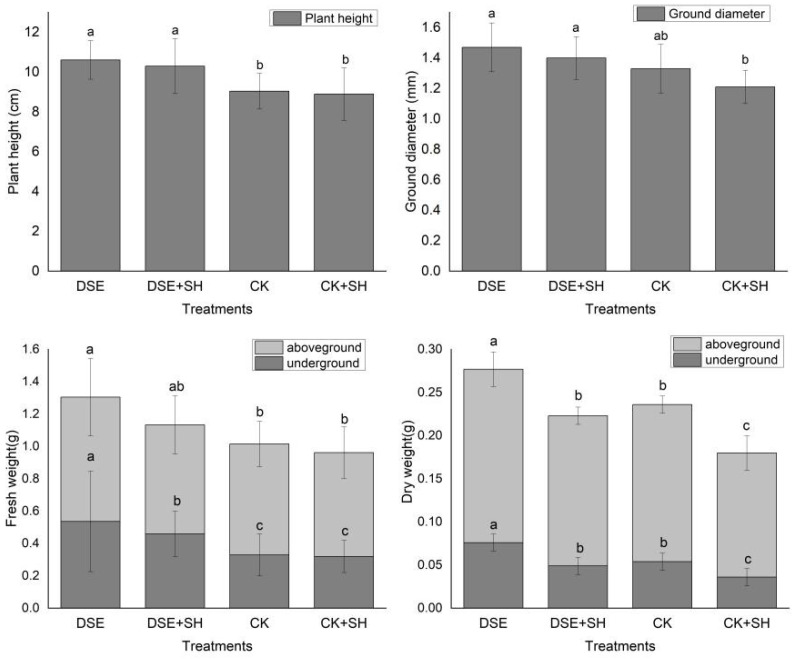
Effects of *P. bamuru* A024 inoculation on plant growth. CK: inoculation with cottonseed shell blank culture medium. DSE: single inoculation with *P. bamuru* A024. CK + SH: Single inoculation with *R. solani* SH01. DSE + SH: sequential inoculation with *P. bamuru* A024 and *R. solani* SH01. Different letters in the columns indicate significant differences (*p* < 0.05), according to a Tukey test.

Dark septate endophytes strain, *P. bamuru* A024 inoculation, accelerated seedling growth (Figure 1), as observed based on significant differences in height between different treatments and control (*p* < 0.05). However, the difference between DSE treatment and DSE + SH treatment showed no significance (*p* ≥ 0.05). The seedling heights under DSE and DSE + SH treatments were increased by 17.31% and 13.94%, separately, compared with control treatment.

#### 3.2.2. Seedling Diameter

Differences in seedling diameter among DSE, DSE + SH, and CK treatment groups were not significant (*p* ≥ 0.05). However, differences among DSE, DSE + SH, and CK + SH treatment groups were significant (*p* < 0.05). Seedling diameters under DSE and DSE + SH treatments were increased by 21.49% and 15.71%, separately, compared with CK + SH treatment.

#### 3.2.3. Seedling Biomass

##### Fresh weight

There were significant differences between DSE, DSE + SH groups, and CK, CK + SH groups (*p* < 0.05) for the seedlings’ fresh underground weight. The fresh underground weight of seedlings from DSE and DSE + SH treatments was increased by 62.91% and 39.51% compared to the CK treatment, respectively. For the above-ground fresh weight, differences among DSE, DSE + SH, CK, CK + SH groups were significant. The fresh above-ground weight of seedlings from DSE treatment increased by 12.09% compared to CK treatment.

##### Dry weight

There were significant differences in both the under- and above-ground dry weights of seedlings between all the treatments. The under- and above-ground dry weight of DSE treatment increased by 40.74% and 10.44% compared to the CK treatment, respectively.

### 3.3. DSE Strain Inoculation Effect on Seedling Root Structures

**Figure 2:**
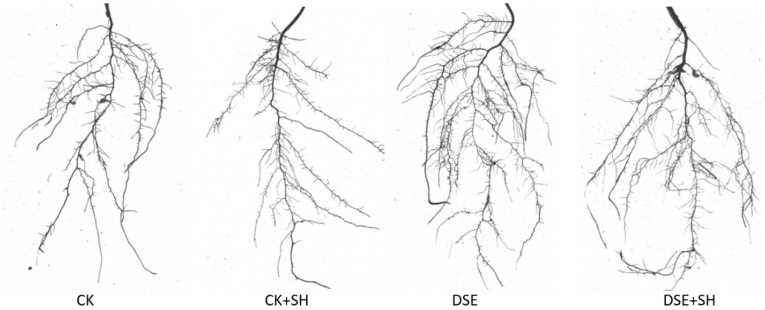
Different inoculation impacts on the root structures of seedlings of CK, CK + SH, DSE, DSE + SH. CK: cottonseed shell blank culture medium inoculation. DSE: *P. bamuru* A024 inoculation alone. CK + SH: Single inoculation with *R. solani* SH01. DSE + SH: sequential inoculation with *P. bamuru* A024 and *R. solani* SH01.

Several parameters, such as surface area and root length, play vital roles in determining root distribution. In contrast, tip number, fork number, root volume and average root diameter exert vital parts in determining the absorption efficiency of roots. It can be found from the data listed in Table 2 and illustrated in Figure 2 that inoculation with *P. bamuru* A024 dramatically enhanced the root system parameters including root surface area, fork number, root length and volume in comparison with control (*p* < 0.05). 

The differences in the above indexes showed no significance between DSE and DSE + SH treatments, except for the tip number index. This result indicates that *R. solani* made no obvious difference to *P. sylvestris* var. *mongolica* root structure after *P. bamuru* A024 inoculation. However, there were significant differences after the CK and CK + SH treatment for all root structure indices except average diameter, indicating that a single inoculation with *R. solani* has a significant impact on the root structure. In addition, the root index values in CK + SH treatment were lower than that of CK group. In comparison with CK group, DSE and DSE + SH groups showed increased root length by 19.13% and 15.53%, root surface area by 17.41%, and 22.29%, root volume by 17.98% and 24.25%, root forks number by 16.34% and 33.03%, respectively.

### 3.4. DSE Inoculation Impacts on the Rhizosphere Soil Physicochemical Characteristics in the Seedlings

It is observed from Table 3 that differences among four treatments were significant (*p* < 0.05). DSE treatment significantly increased the soil nutrient content compared to CK treatment. The soil nutrient index includes OM, TN, AN, TP, AP, TK, AK, that increased by 10.96%, 12.16%, 57.57%, 32.53%, 103.84%, 33.11% and 23.52%, respectively. *R. solani* inoculation (DSE + SH treatment) decreased the effect of DSE treatment of raising the nutrient index. However, compared to the CK + SH treatment, the soil nutrient index that includes TP, AP, and AK increased by 15.07%, 34.61%, and 32.35%, respectively. The effect of DSE inoculation on AN, AP, and AK improvement was more significant than TN, TP, and TK. It is possibly because that DSE can degrade the macromolecular nutrients in soils to the effective state to be used for plants, which thus accelerates the energy flow and nutrient recycling in soil.

### 3.5. DSE Inoculation Impacts on Enzyme Activities in the Rhizosphere Soil

The soil-borne enzymes that make up the organic components with the highest activity of biochemical processes of soil mostly come from secretions by animals, plants, or soil microorganisms. They exert a vital role in circulating soil OM and conserving energy.

According to Table 3, *P. bamuru* A024 inoculation significantly elevated several enzyme activities in seedling rhizosphere soil. In comparison to CK treatment, activities of CAT, urease, and acid phosphatase under DSE treatment increased by 12.97%, 20.93% and 19.67%, respectively. Additionally, catalase, urease, and protease of soil from DSE + SH treatment increased by 16.64%, 20.22%, and 38.57%, respectively. As suggested by the above findings, *P. bamuru* A024 exerted a vital role in soil energy flow and nutrient circulation, which also significantly promoted the nutrient circulation and activities of soil enzymes.

Pearson’s analysis assessed the correlations of biomass index with soil physicochemical characters (Table 4). TN indices showed a significant positive correlation with the total dry weight and the fresh underground weight. TP showed a significant positive correlation with above-ground dry weight, fresh underground weight, total dry weight, and underground dry weight. AP displayed a significant positive correlation with total dry weight, whereas the AK displayed a significant positive correlation with underground dry weight and total dry weight.

### 3.6. Seedling Rhizosphere Bacterial Diversity

#### 3.6.1. Sequencing Results for Soil Samples and Validation of Sampling Depth

**Figure 3:**
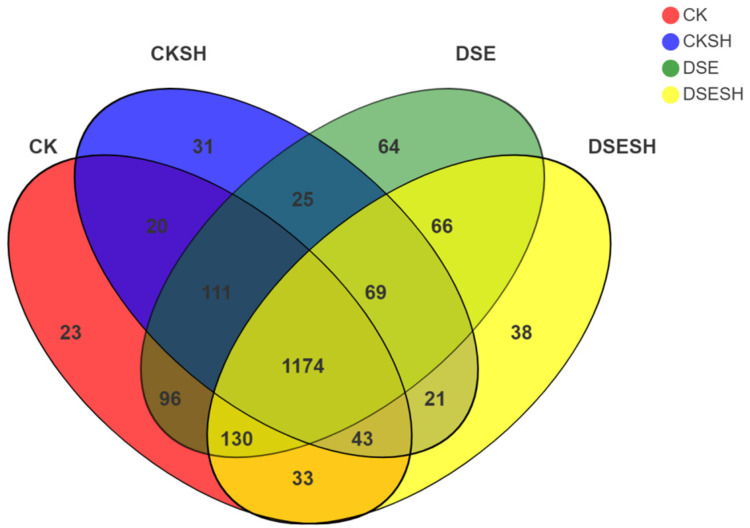
Venn diagram presenting the common OTUs under CK, CK + SH, DSE, DSE + SH treatments. CK: cottonseed shell blank culture medium inoculation. DSE: *P. bamuru* A024 inoculation alone. CK + SH: Single inoculation with *R. solani* SH01. DSE + SH: sequential inoculation with *P. bamuru* A024 and *R. solani* SH01.

Altogether 444,738 bacterial sequences were acquired based on twelve blended soil samples from the four treatment groups by using the Illumina MiSeq PE300 platform. On the whole, following isolation and removal, this study acquired 1944 bacterial OTUs from clustering, with the similarity threshold of 97%. Those representative bacterial OTUs for DSE, DSE + SH, CK, as well as CK + SH treatments were 64, 38, 23, and 31, separately. In addition, DSE, DSE + SH, CK, and CK + SH treatments held 1174 common OTUs; whereas CK and DSE treatments held 1752 common OTUs, and DSE and DSE + SH treatments held 1638 OTUs (Figure 3).

#### 3.6.2. Soil Microbial Distribution

According to categorical analysis on the typical OTUs sequences at a similarity threshold of 97%, altogether 32 phylum, 32 classes, 72 orders, 137 families, 252 genera, and 448 species of soil bacteria were detected. As showed in Table 5, the main bacterial phyla within the rhizosphere soil of the seedlings of *P. sylvestris* var. *mongolica* include Proteobacteria, Saccharibacteria, Actinobacteria, Gemmatimonadetes, Bacteroidetes, Chloroflexi, Acidobacteria, Firmicutes, Parcubacteria, Verrucomicrobia, Planctomycetes, and Cyanobacteria. Among them, the relative abundance of the Proteobacteria phylum was highest in all four treatments, CK, CK + SH, DSE, and DSE + SH, which were 44.01%, 39.55%, 42.63%, and 45.92%, respectively. There was no significant difference between the four treatments and the relative abundance of Proteobacteria, Acidobacteria, Parcubacteria, Verrucomicrobia, Planctomycetes, and Cyanobacteria phylum. The relative abundance of the Actinobacteria, Gemmatimonadetes were higher in CK and CK + SH treatments, whereas Bacteroidetes, Chloroflexi and Firmicutes were higher in DSE and DSE + SH treatments.The bacterial phylum with significant differences in relative abundance, that is, Gemmatimonadetes and and Actinobacteria were found higher in CK (10.78%) and CK + SH (14.32%) treatments. In contrast, Saccharibacteria (15.11%), Bacteroidetes (8.30%), and Chloroflexi (5.28%) were higher in DSE treatment, and Firmicutes were higher in DSE + SH treatment with 6.91%. The difference between the major bacterial phylum in different treatments indicated that DSE inoculation had little effect on the Proteobacteria phylum with the highest relative abundance but has a significant impact on the relative abundance of some other major bacterial phylum.

Pearson’s analysis assessed the correlations of bacterial phylum with the physicochemical characters in soil (Table 6). Four bacterial phyla, including Actinobacteria, Gemmatimonadetes, Bacteroidetes, and Firmicutes, were significantly correlated with soil physicochemical properties. Actinobacteria showed a significant negative correlation with TN, TP, AP, AK and OM and a significant positive correlation with TK. Bacteroidetes displayed a significant positive correlation with OM and AK, while Firmicutes showed a significant positive correlation with AP, TN and TP, but significant negative correlation with TK. Bacteroidetes and Firmicutes had the highest relative abundance in DSE treatment compared to other treatments. Conversely, Actinobacteria and Gemmatimonadetes had a higher relative abundance in CK treatment than other treatments. 

This indicated that after dark septate endophytes inoculation, the bacterial phylum that showed an increase in its relative abundance has a positive effect on improving the rhizosphere nutrients of *Pinus sylvestris* var. *mongolica*.

**Figure 4:**
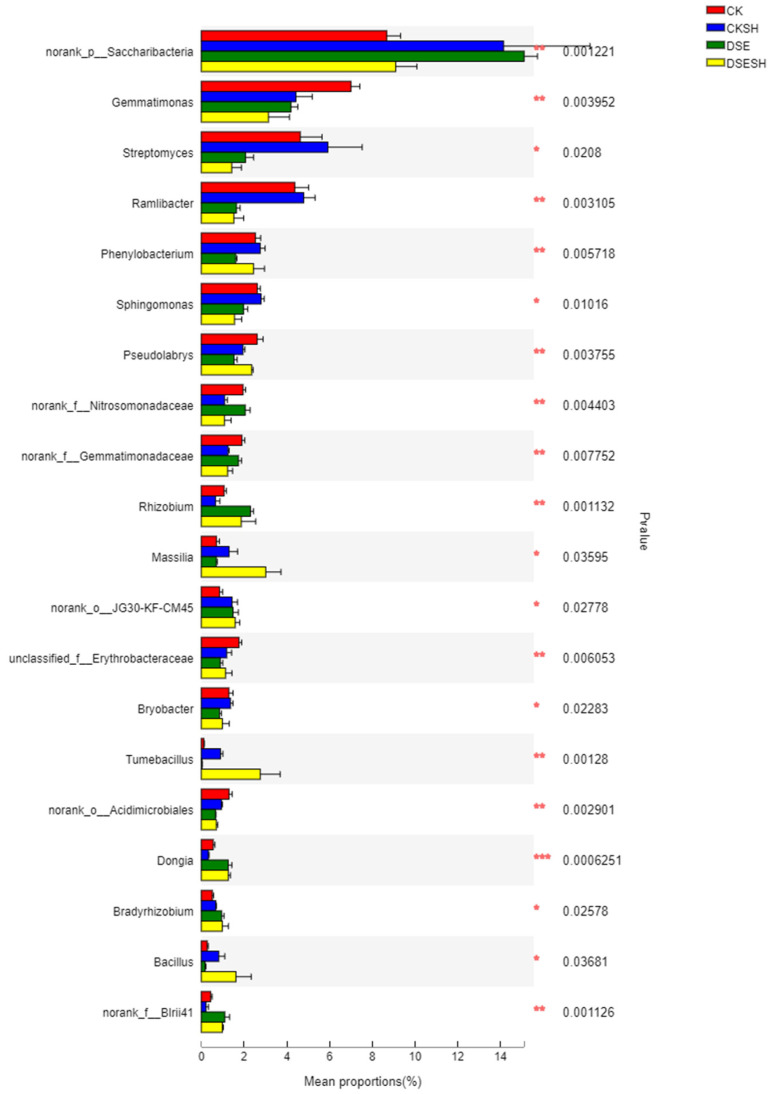
One-way ANOVA bar plot showing the significant differences in bacterial genera in CK, CK + SH, DSE, DSE + SH treatments. CK: cottonseed shell blank culture medium inoculation. DSE: *P. bamuru* A024 inoculation alone. CK + SH: Single inoculation with *R. solani* SH01. DSE + SH: sequential inoculation with *P. bamuru* A024 and *R. solani* SH01. * *p* < 0.05 (both sides).

This study identified altogether 252 bacterial genera under four treatments. One-way ANOVA analysis was used for testing the significant difference between bacterial genera and diverse treatments, followed by the post-hoc test to find the sample groups with differences in the multiple groups (Figure 4). The analysis of the top 20 genera with significant differences showed that DSE treatment could change relative abundances for rhizosphere bacterial genera. Typically, relative abundances of *norank_p_Saccharibacteria*, *Massilia*, *Rhizobium*, *Bacillus*, *Tumebacillus, Dongia, Norank_f_Blrii41* and *Bradyrhizobium* were higher in DSE treatments (DSE and DSE + SH), in contrast, the relative abundance of *Streptomyces*, *Gemmatimonas*, *Ramlibacter, Sphingomonas*, *Bryobacter*, *norank_o_Acidimicrobiales* was higher in CK treatments (CK and CK + SH).

#### 3.6.3. α-Diversity Analysis

ANOVA was carried out on 16S rDNA diversity indexes for soil samples under CK, CK + SH, DSE, and DSE + SH treatments (Table 7). According to Table 3, although those coverage indices under K, CK + SH, DSE, and DSE + SH treatments approached 1, no significant difference was found. Based on the above results, our sequencing results might be used to precisely represent the real soil samples conditions. As for Chao1 and Ace indices, they followed the orders of DSE > CK and DSE + SH > CK + SH under different treatments, and apparent differences were detected under CK treatment compared with the other three treatments (*p* < 0.05). This indicates increased total bacterial community number as well as richness under the DSE treatment relative to the others, including CK treatment. Differences in Simpson index among CK, CK + SH, DSE, and DSE + SH groups were not significant. As for the Shannon index, it followed the order of DSE > CK, CK + SH, and DSE + SH, and differences of DSE were significant compared with other three treatments. These results showed that DSE significantly affected rhizosphere soil bacterial richness of *P. sylvestris* var. *mongolica*, but less on diversity.

#### 3.6.4. β-Diversity Analysis

**Figure 5:**
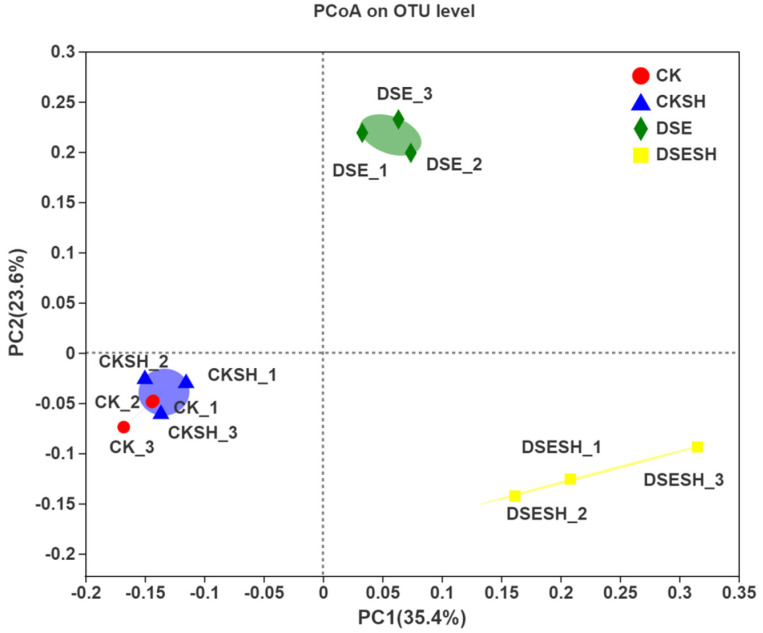
PCoA ordination according to the Bray-Curtis similarities for bacterial communities in CK, CK + SH, DSE, DSE + SH treatments. CK: inoculation with cottonseed shell blank culture medium. DSE: single inoculation with *P. bamuru* A024. CK + SH: Single inoculation with *R. solani* SH01. DSE + SH: sequential inoculation with *P. bamuru* A024 and *R. solani* SH01.

Differences in bacterial communities of different soil samples were measured through the Bray–Curtis matrix. According to Figure 5, CK and CK + SH, DSE, and DSE + SH groups were divided into three parts, which showed distribution among diverse quadrants. The great distribution distance represented the great differences in composition in the three parts that included CK and CK + SH, DSE, DSE + SH samples. As figured out from this result, there were more differences in soil bacterial communities among CK and CK + SH, DSE, and DSE + SH treatments than those under each treatment (*R* = 1), with significant differences (*p* = 0.003).

#### 3.6.5. Environmental Factor Correlation Analysis

**Figure 6:**
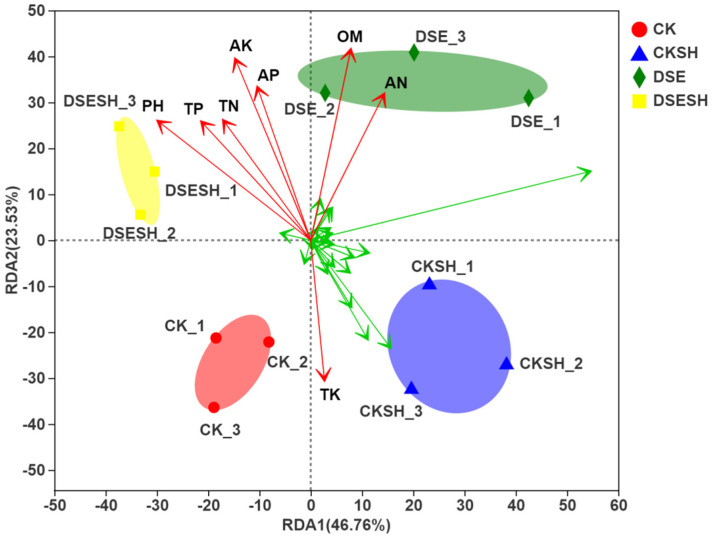
Environmental Factor Correlation Analysis of a redundancy analysis (RDA) of MiSeq data (symbols and green arrows) and environmental characteristics (red arrows) in CK, CK + SH, DSE, DSE + SH treatments. CK: inoculation with cottonseed shell blank culture medium. DSE: single inoculation with *P. bamuru* A024. CK + SH: Single inoculation with *R. solani* SH01. DSE + SH: sequential inoculation with *P. bamuru* A024 and *R. solani* SH01. OM: Organic matter. TN: Total nitrogen. AN: Available nitrogen. TP: Total phosphorus. AP: Available phosphorus. TK: Total potassium. AK: Available potassium. pH: soil pH.

DSE inoculation changes the bacterial community structures and environmental characteristics. Soil geochemical characteristics may primarily mediate the effect of DSE inoculation on bacterial communities. Therefore, this study investigated whether bacterial community structure and ecological characteristics are related. Redundancy analysis (RDA) was carried out for 30 bacterial genera with significant differences and the environmental variables that include soil pH, TN, OM, TP, TK, AK, AP, and AN. It is shown in Figure 6 that RDA was carried out to assess those associations of bacterial community structure with a variety of environmental factors by displaying samples in scattered form, quadrant distribution, and the arrow direction and length. RDA analysis showed that 70% of the information regarding the community structure were interpreted using eight environmental factors, among which 46.76% of the variation was explained by RDA1 and 23.53% by RDA2. RDA revealed that the primary environmental characteristics formed the microbial community structure. The environmental factors that showed a high correlation with the composition of the bacterial community of DSE and DSE + SH treatments soil samples were AN, OM, AP, AK, TN, TP, and pH. The environmental factor that showed a high correlation with the composition of the bacterial community structure of CK and CK + SH treatments soil samples was TK. In addition, the environmental factors AN, OM, AP, AK, TN, TP, pH were positively correlated with each other.

## 4. Discussion

The plant rhizosphere is a complex micro-ecological system wherein the interaction between beneficial microorganisms and host plants can promote the growth and stress resistance of plants. Dark septate endophytes (DSEs) can colonize the cortex and epidermis spaces inside and outside cells, or even the vessel tissue of healthy plant roots, to form symbionts. DSEs play an important part in promoting plant development and enhancing nutrient absorption, along with stress resistance. Strain A024 isolated and screened from the roots of *P. sylvestris* var. *Mongolica* was found to effectively suppress *R. solani* in vitro (unpublished data). To further study the interaction mechanism between the strain A024 and *P. sylvestris* var. *mongolica* and its control effect on *R. solani*, we have mainly focused on: (1) The control effect of strain A024 on *R. solani*; (2) the effect on promoting *P. sylvestris* var. *mongolica*; (3) the impacts on microbial community structure in rhizosphere soil.

This study reported a damping-off rate of 28.15% and a survival rate of 80.15% for DSE + SH treatment. Whereas for CK + SH treatment, the incidence rate of damping-off was 72.15%, and the survival rate was 33.35%. However, the relative control effect of strain A024 treatment against damping-off was 60.98%. These results of the seedling inoculation test showed that *P. bamuru* A024 could effectively control the occurrence of damping-off caused by *R. solani* on *P. sylvestris* var. *mongolica* seedlings. These results are similar to those of other *Phialocephala* studies. Christoph [34] isolated 85 PAC strains from Norway spruce (*Picea abies*) roots, among which *P. europaea* dramatically decreased *Phytophthora citricola* growth in vitro. The main inhibitory agents were identified as sclerotinin A and B, sclerolide, and sclerin, however, no seedling inoculation test was done. The result of the sterile seedlings inoculation experiment done by Terhonen indicated that *P. sphareoides* could prevented Norway spruce seedling root infection by the pathogen, *Heterobasidion parviporum* under *in-vitro* conditions. These results indicated that DSE fungi could colonize the host root system and effectively inhibit the occurrence of soil-borne diseases. Beside this, DSE fungi can also enhance seedlings’ disease resistance against soil borne pathogens. In our research, under the stress of *R. solani*, the β–1,3-glucanase and chitinase activity, together with the contents of proline and soluble protein in seedlings from DSE + SH treatment increased by 18.41%, 92.54%, 101.27% and 30.48% respectively in comparison to the CK + SH treatment (Table 1). Su [41] found that the DSE strain *Harpophora oryzae* enhanced disease resistance in rice and reduced root blast disease caused by *Magnaporthe oryzae* by up-regulation of expression of key genes of salicylic acid (SA) signaling pathway. Therefore, improving plant resistance is one of the main reasons to reduce the occurrence of soil-borne disease.

Dark septate endophytes (DSE) also has an effect on plant growth. The direct effects include promoted plant growth, an increase in biomass, as well as boosting the root structure and development of plants. In our research, dark septate endophytes strain A024 enhanced annual seedling growth, compared to CK treatment, underground and aboveground fresh weight, seedling height, aboveground and underground dry weight of seedlings from DSE treatment increased by 17.31%, 62.91%, 12.09%, 40.74%, 10.43%, respectively. Similarly, fresh underground weight, ground diameter, seedling height, underground and aboveground dry weights under DSE + SH treatment increased by 15.82%, 15.71%, 43.43%, 36.11%, 36.11%, and 20.83%, respectively, compared to CK + SH treatment (Figure 1). These results indicated that DSE has an effect on plant growth even under the stress of *R. solani*. Andrade [42] inoculated tomato plants with *Leptodontidium orchidicola* liquid inoculum, that increased the biomass by 20%, and number of fruits by two-fold, in comparison with control. The same result was found in an experiment that involved inoculation of *Harpophora radicicola* with *Vulpia ciliate* [43]. DSE fungi can secrete a variety of extracellular hydrolases, such as amylase, pectinase, laccase, cellulase, lipase, protease, tyrosinase, polyphenol oxidase, and xylanase. Thus, the presence of a variety of hydrolases ensures the utilization of various nutrients [44]. Dark septate root endophytic fungi, *Phialocephala fortinii,* increased the growth of the seedlings for Scots pine in the presence of increased CO_2_ content via enhancing the utilization efficiency of nitrogen [45]. Surface area and root length account for the vital factors to measure root distribution, meanwhile, root volume, average root diameter, fork number and tip number are also the vital factors to measure the absorption efficiency of root. Inoculation with DSEs can regulate the plant root architecture and facilitate the growth of root systems. In our research, DSE A024 significantly (*p* < 0.05) enhanced root volume and length, fork number and root surface area in *P. sylvestris* var. *mongolica* in comparison with control treatment. Although, differences in the root architecture indexes were not significant between DSE and DSE + SH treatments, but CK and CK + SH treatments showed a significant difference (Table 2 and Figure 2). These results showed that DSE inoculation could counteract the negative effect of the pathogen on the root structure. The change of root structure promoted the growth of annual seedlings.These results are similar to other studies, Li [46] inoculated *Leptosphaeria* sp., *knufia* sp., and *Darksidea* sp., with *A. mongolicus*, and the plant root biomass and length remarkably elevated compared to control. *P. graminicola* inoculation can also change the specific root length, root diameter, and root hair number of *Vulpia ciliata* ssp. *Ambigua* [47].

A number of soil nutrient elements show low or no solubility, thus limiting soil nutrient circulation [48,49]. The existence of P and N within soil limits absorption and utilization efficiency of plants. Conversion of these organic nutrients to the inorganic ones to be easily taken up by plants depends on the microbes-derived extracellular enzymes, such as bacteria or fungi. DSE can improve the efficiency of plant uptake and utilization of soil nutrients. In our research, the seedlings of *P. sylvestris* var. *mongolica*, rhizosphere soil nutrient indices including AN, TP, AP, TK, AK increased by 57.57%, 32.53%, 103.84%, 33.11%, and 23.52%, respectively under the DSE treatment compared to the CK treatment (Table 3). Soil enzymes play a vital part in conserving energy and circulating organic matter in soil. *P. bamuru* A024 inoculation significantly elevated enzyme activities in seedling rhizosphere soil. In comparison to CK treatment, activities of catalase, urease, and acid phosphatase under DSE treatment increased by 12.97%, 20.93% and 19.67%, respectively (Table 3). Therefore, DSE can improve the content of soil available K and P, thereby improving plant absorption efficiency while increasing the accumulation of biomass. In other research, Jumpponen et al. combining DSE inoculation with nitrogen fertilizer application increased the biomass accumulation of seedlings by 50% compared to the application of nitrogen fertilizer alone, which proved that the existence of DSE improved the utilization efficiency of the nitrogen fertilizer [50]. The inoculation of *Atriplex canescens* (Pursh) Nutt. with *Aspergillus ustus* increased the P utilization efficiency, especially for the unavailable P [51]. Spagnoletti measured the ability of nine DSE strains for solubilizing aluminum and iron phosphates, and DSE could solubilize insoluble phosphate [52]. Knapp [53] used Biolog FF together with API-ZYM tests for analyzing 15 DSE’s metabolic diversity. Differences were detected across diverse species, but each of our tested substrate was absorbed by DSE fungi. So the improvement of soil nutrients is one of important reasons to seedlings growth promotion.

According to our high-throughput sequencing analysis results, the rhizosphere bacterial community of *P. sylvestris* var. *mongolica* is dominated by Proteobacteria, with the highest relative abundance among the four treatments. However, the differences between the four treatments are not significant. The relative abundance of the Actinobacteria, Gemmatimonadetes were higher in CK and CK + SH treatments, whereas Bacteroidetes, Chloroflexi and Firmicutes were higher in DSE and DSE + SH treatments (Table 5). The results of α-diversity index analysis showed that DSE had a significant effect on rhizosphere soil richness for *P. sylvestris* var. *mongolica*, but little effect on the diversity. Moreover, it was found through analyzing β-Diversity Indexes that, there were higher differences between bacteria groups under CK and CK + SH, DSE, and DSE + SH treatments than those inside each treatment (Figure 5). And some genus include *Massilia*, *Rhizobium*, *Bacillus*, *Tumebacillus*, *Dongia* and *Bradyrhizobium* were higher in DSE treatments, in contrast, the relative abundance of *Streptomyces*, *Gemmatimonas*, *Ramlibacter*, *Sphingomonas* and *Bryobacter* were higher in CK treatments (Figure 4). And the RDA analysis result indicated that DSE treatments soil bacterial community structure were positive correlated with AN, OM, AP, AK, TN, TP (Figure 6), and this mean after DSE inoculation, enhance the soil nutrition, and then promote the growth of annual seedlings.

In conclusion, dark septate endophyte (DSE) A024 can control the rate of incidence of seedling damping-off disease and promote annual seedling growth after inoculation, and change the root structure of the *P. sylvestris* var. *Mongolica* seedlings, thereby elevating the seedling growth potential as well as absorption area. DSE A024 increased the activities of soil enzymes and contents of soil nutrients in the rhizosphere soil of *P. sylvestris* var. *mongolica*., particularly, the rhizosphere soil bacterial community structure of DSE A024 inoculation was positively correlated to the main soil nutrition indices.

## Figures and Tables

**Table 1:** Effects of different inoculation treatments on seedling physiological indices.

Treatment	β-1,3-Glucanase Level (U/g)	Chitinase Level (U/g)	SOD Level (U/g)	CAT Level (U/g)	POD Level (U/g)
CK	12.97 ± 1.13c	57.81 ± 1.90d	570.57 ± 33.58a	75.90 ± 4.99c	443.94 ± 4.07b
DSE	13.98 ± 1.65c	94.35 ± 4.57c	615.34 ± 21.69a	121.02 ± 10.19a	1108.22 ± 19.44a
CK + SH	23.84 ± 2.56b	124.84 ± 12.03b	586.80 ± 55.06a	94.76 ± 2.45bc	480.61 ± 17.68b
DSE + SH	28.23 ± 0.76a	240.37 ± 5.43a	658.91 ± 18.12a	105.14 ± 9.62ab	520.25 ± 6.72b
**Treatment**	**Soluble Protein** **Level (mg/L)**	**Proline Level (ug/g)**	**MDA** **Level nmol/g**	**Soluble Sugar** **Level (mg/g)**	**PAL** **Level (U/g)**
CK	51.73 ± 6.07b	41.01 ± 2.97c	24.99 ± 1.62a	36.78 ± 3.81a	24.21 ± 0.99b
DSE	77.33 ± 3.29a	52.83 ± 1.21bc	25.99 ± 1.77a	38.34 ± 5.57a	27.70 ± 1.64b
CK + SH	60.07 ± 3.78b	58.69 ± 3.58b	21.68 ± 1.78a	30.93 ± 8.95a	36.03 ± 1.55a
DSE + SH	78.38 ± 5.25a	118.13 ± 9.01a	34.20 ± 1.10a	38.94 ± 4.50a	38.61 ± 2.12a

CK: cottonseed shell blank culture medium inoculation. DSE: *P. bamuru* A024 inoculation alone. CK + SH: Single inoculation with *R. solani* SH01. DSE + SH: sequential inoculation with *P. bamuru* A024 and *R. solani* SH01. MDA: Malonaldehyde. SOD: Superoxide dismutase. POD: Peroxidase. CAT: Catalase. PAL: Phenylalanine ammonia lyase. Different letters in the columns indicate significant differences (*p* < 0.05), according to a Tukey test.

**Table 2:** *DSE* inoculation effect on the root structures of seedlings.

Index	CK	CK + SH	DSE	DSE + SH
Root length/cm	170.16 ± 11.68b	140.34 ± 10.05c	202.72 ± 10.35a	196.59 ± 11.32a
Root surface area/cm^2^	27.829 ± 2.01b	22.605 ± 1.89c	32.675 ± 2.09a	34.034 ± 3.33a
Average diameter/mm	0.523 ± 0.005a	0.513 ± 0.022a	0.526 ± 0.008a	0.538 ± 0.010a
Tips number	272.12 ± 13.35b	260.87 ± 16.35c	275.90 ± 13.42b	356.10 ± 15.05a
Root Volume(cm^3^)	0.367 ± 0.011b	0.289 ± 0.006c	0.433 ± 0.033a	0.456 ± 0.025a
Forks number	357.12 ± 10.78b	278.12 ± 7.01c	415.50 ± 10.36a	475.10 ± 11.91a

CK: cottonseed shell blank culture medium inoculation. DSE: *P. bamuru* A024 inoculation alone. CK + SH: Single inoculation with *R. solani* SH01. DSE + SH: sequential inoculation with *P. bamuru* A024 and *R. solani* SH01. Diverse letters within each column are indicative of significant differences (*p* < 0.05) upon Tukey test.

**Table 3:** Different inoculation treatment impacts on the enzyme activities and nutrients in soil.

Index	CK	CK + SH	DSE	DSE + SH
OM g/kg	114.03 ± 0.21d	115.83 ± 0.35c	126.53 ± 0.88a	120.97 ± 0.21b
TN g/kg	7.73 ± 0.199bc	7.39 ± 0.056c	8.67 ± 0.028a	8.03 ± 0.298b
AN mg/kg	0.33 ± 0.007d	0.47 ± 0.005c	0.52 ± 0.001a	0.49 ± 0.003b
TP g/kg	1.26 ± 0.049c	1.17 ± 0.028d	1.67 ± 0.049a	1.36 ± 0.026b
AP mg/kg	0.78 ± 0.053d	1.06 ± 0.013c	1.59 ± 0.005a	1.33 ± 0.014b
TK g/kg	9.06 ± 0.088d	9.85 ± 0.014c	12.06 ± 0.248a	10.32 ± 0.073b
AK mg/kg	0.34 ± 0.033b	0.32 ± 0.023c	0.42 ± 0.015a	0.43 ± 0.014a
pH value	5.65 ± 0.011b	5.62 ± 0.015b	5.51 ± 0.021c	5.71 ± 0.055a
Sucrase level U/g	42.99 ± 1.69a	39.82 ± 5.67a	41.97 ± 3.34a	44.46 ± 1.22a
Catalase level U/g	24.81 ± 1.58b	23.73 ± 0.82b	27.89 ± 2.50a	27.68 ± 2.45a
Acid phosphatase levelU/g	373.70 ± 18.25b	365.62 ± 15.59b	445.63 ± 16.51a	358.44 ± 9.03b
Urease level U/g	231.66 ± 9.14b	219.49 ± 2.43b	277.62 ± 15.80a	263.88 ± 8.06a
Protease U/g	20.16 ± 4.75b	20.56 ± 0.37b	22.13 ± 4.37b	28.49 ± 3.24a

CK: cottonseed shell blank culture medium inoculation. DSE: *P. bamuru* A024. CK + SH inoculation alone: Single inoculation with *R. solani* SH01. DSE + SH: sequential inoculation with *P. bamuru* A024 and *R. solani* SH01. OM: Organic matter. TN: Total nitrogen. AN: Available nitrogen. TP: Total phosphorus. AP: Available phosphorus. TK: Total potassium. AK: Available potassium. pH: soil pH. The diverse letters within each column are indicative of significant differences (*p* < 0.05) upon Tukey test.

**Table 4:** The correlations of seedling biomass with soil physicochemical characters.

Index	Height	Diameter	Fresh Weight			Dry Weight		
			Aboveground	Undergound	Total	Aboveground	Undergound	Total
OM	-	-	-	-	-	-	-	-
TN	-	-	-	0.587 *	-	-	-	0.686 *
AN	-	-	-	-	-	-	-	-
TP	-	-	-	0.602 *	-	0.587 *	0.579 *	0.762 **
AP	-	-	-	-	-	-	-	0.646 *
TK	-	-	-	-	-	-	-	-
AK	-	-	-	-	-	-	0.619*	0.605 *

OM: Organic matter. TN: Total nitrogen. AN: Available nitrogen. TP: Total phosphorus. AP: Available phosphorus. TK: Total potassium. AK: Available potassium. ** Indicator is significantly correlated at level 0.01 (both sides), * indicator is significantly correlated at level 0.05 (both sides), - indicates no significant correlation.

**Table 5:** The relative abundance of the main phylum in CK, CK + SH, DSE, DSE + SH treatments.

Treatment	Proteobacteria	Saccharibacteria	Actinobacteria	Ge mMatimonadetes	Bacteroidetes	Chloroflexi
CK	44.01 ± 3.55a	8.68 ± 0.66c	12.92 ± 0.37ab	10.78 ± 0.85a	6.20 ± 0.16ab	4.65 ± 0.99b
CK + SH	39.55 ± 2.09a	14.15 ± 1.04b	14.32 ± 0.43a	8.09 ± 0.78ab	5.00 ± 0.19b	4.21 ± 0.30b
DSE	42.63 ± 2.86a	15.11 ± 0.63a	8.91 ± 1.08bc	7.50 ± 0.59b	8.30 ± 1.27a	5.28 ± 0.38a
DSE + SH	45.92 ± 1.54a	9.10 ± 0.99c	9.99 ± 0.94b	5.77 ± 0.42b	6.53 ± 0.45ab	4.92 ± 0.24b
	**Acidobacteria**	**Firmicutes**	**Parcubacteria**	**Verrucomicrobia**	**Planctomycetes**	**Cyanobacteria**
CK	5.60 ± 0.16a	1.16 ± 0.05b	1.55 ± 0.13a	1.65 ± 0.07a	0.68 ± 0.01a	0.43 ± 0.02a
CK + SH	4.11 ± 0.27a	2.78 ± 0.11b	2.52 ± 0.02a	1.72 ± 0.01a	1.07 ± 0.05a	0.91 ± 0.05a
DSE	4.68 ± 0.66a	0.91 ± 0.05b	2.02 ± 0.05a	1.66 ± 0.09a	0.44 ± 0.01a	0.64 ± 0.01a
DSE + SH	4.07 ± 0.11a	6.91 ± 0.24a	1.82 ± 0.02a	2.12 ± 0.04a	0.55 ± 0.02a	0.88 ± 0.03a

CK: cottonseed shell blank culture medium inoculation. DSE: *P. bamuru* A024 inoculation alone. CK + SH: Single inoculation with *R. solani* SH01. DSE + SH: sequential inoculation with *P. bamuru* A024 and *R. solani* SH01. The diverse letters within each column are indicative of significant differences (*p* < 0.05) upon Tukey test.

**Table 6:** Correlations of Rhizosphere Soil Physicochemical Properties with bacterial phylum.

Index	Actinobacteria	Gematimonadetes	Bacteroidetes	Firmicutes
OM	−0.834 **	-	0.698 *	-
TN	−0.578 *	−0.803 **	-	0.736 **
AN	-	−0.797 **	-	-
TP	−0.588 *	−0.813 **	-	0.806 **
AP	−0.667 *	−0.873 **	-	0.671 *
TK	-	0.888 **	-	−0.634 *
AK	−0.889 **	−0.640 *	0.651 *	-

OM: Organic matter. TN: Total nitrogen. AN: Available nitrogen. TP: Total phosphorus. AP: Available phosphorus. TK: Total potassium. AK: Available potassium. ** *p* < 0.01 (both sides), * indicator *p* < 0.05 (both sides), - indicates no significant correlation.

**Table 7:** DSE inoculation impacts on soil bacterial community diversity under CK, CK + SH, DSE, DSE + SH treatments.

Samples	ACE Index	Chao1 Index	Shannon Index	Simpson Index	Coverage %
CK	1558.89 ± 21.68b	1559.67 ± 32.07b	5.85 ± 0.11a	0.0073 ± 0.0004a	0.99 ± 0.0a
CK + SH	1401.89 ± 83.79c	1403.51 ± 85.19c	5.42 ± 0.27b	0.015 ± 0.00078a	0.99 ± 0.0a
DSE	1668.45 ± 17.38a	1680.85 ± 10.42a	5.80 ± 0.03ab	0.0124 ± 0.0009a	0.99 ± 0.0a
DSE + SH	1496.54 ± 24.78bc	1517.02 ± 32.11bc	5.77 ± 0.05ab	0.0086 ± 0.0004a	0.99 ± 0.0a

CK: inoculation with cottonseed shell blank culture medium. DSE: single inoculation with *P. bamuru* A024. CK + SH: Single inoculation with *R. solani* SH01. DSE + SH: sequential inoculation with *P. bamuru* A024 and *R. solani* SH01. Different letters in the columns indicate significant differences (*p* < 0.05), according to Tukey test.
